# The application value of multi-parameter cystoscope in improving the accuracy of preoperative bladder cancer grading

**DOI:** 10.1186/s12894-022-01054-z

**Published:** 2022-07-18

**Authors:** Qikai Wu, Lingkai Cai, Baorui Yuan, Qiang Cao, Juntao Zhuang, Meiling Bao, Zhen Wang, Dexiang Feng, Jun Tao, Pengchao Li, Qiang Shao, Xiao Yang, Qiang Lu

**Affiliations:** 1 Department of Urology, The First Affiliated Hospital of Nanjing Medical University, Nanjing, 210029 People’s Republic of China; 2 Department of Pathology, The First Affiliated Hospital of Nanjing Medical University, Nanjing, 210029 People’s Republic of China; 3 Department of Urology, The Affiliated Suzhou Hospital of Nanjing Medical University, Suzhou Municipal Hospital, Gusu School, Nanjing Medical University, Suzhou, 215008 People’s Republic of China

**Keywords:** Bladder cancer, Cystoscopic biopsy, Pathological grade, Predictive model, High grade

## Abstract

**Purpose:**

To develop and validate a preoperative cystoscopic-based predictive model for predicting postoperative high-grade bladder cancer (BCa), which could be used to guide the surgical selection and postoperative treatment strategies.

**Materials and methods:**

We retrospectively recruited 366 patients with cystoscopy biopsy for pathology and morphology evaluation between October 2010 and January 2021. A binary logistic regression model was used to assess the risk factors for postoperative high-grade BCa. Diagnostic performance was analyzed by plotting receiver operating characteristic curve and calculating area under the curve (AUC), sensitivity, specificity. From January 2021 to July 2021, we collected 105 BCa prospectively to validate the model's accuracy.

**Results:**

A total of 366 individuals who underwent transurethral resection of bladder tumor (TURBT) or radical cystectomy following cystoscopy biopsy were included for analysis. 261 (71.3%) had a biopsy pathology grade that was consistent with postoperative pathology grade. We discovered five cystoscopic parameters, including tumor diameter, site, non-pedicled, high-grade biopsy pathology, morphology, were associated with high-grade BCa. The established multi-parameter logistic regression model (“JSPH” model) revealed AUC was 0.917 (*P* < 0.001). Sensitivity and specificity were 86.2% and 84.0%, respectively. And the consistency of pre- and post-operative high-grade pathology was improved from biopsy-based 70.5% to JSPH model-based 85.2%. In a 105-patients prospective validation cohort, the consistency of pre- and post-operative high-grade pathology was increased from 63.1 to 84.2% after incorporation into JSPH model for prediction.

**Conclusion:**

The cystoscopic parameters based “JSPH model” is accurate at predicting postoperative pathological high-grade tumors prior to operations.

**Supplementary Information:**

The online version contains supplementary material available at 10.1186/s12894-022-01054-z.

## Introduction

Carcinoma of the bladder (BCa) is one of the most common malignancy in urology, with its incidence ranked tenth among malignancies worldwide [1], posing a great challenge in urologic oncology due to its propensity to recur and progress. The histologic grade is important in developing strategies for diagnosing and treating BCa [2], especially the selection of treatment options. Nowadays, transurethral resection of bladder tumor (TURBT) is commonly used for precise preoperative diagnosis. However, TURBT is not without flaws. To begin, it is debatable whether TURBT specimens generated via hot loop resection are fully reliable for histological evaluation[3]. Cheng et al. determined that in 105 matched TURBT patients, the rate of down- and upstaging at the time of RC was 3.8% and 76.2%, respectively [4]. Several studies have also shown that approximately 9–49% of bladder tumors were understaged on histopathological examination, hence increasing the risk of early recurrence and progression [5–8]. Second, TURBT may induce tumor cells to be released into the bloodstream, potentially resulting in potential tumor dissemination [9]. Third, the cost of anesthesia and surgery would impose an extra burden on financially strapped families. Fourth, as a diagnostic technique, TURBT would expose patients to unnecessary risk of anesthesia. Certain patients who are at a high risk of anesthesia may only have one surgical opportunity. As a result, an alternative precise preoperative histologic grade diagnostic approach is urgently needed.

Although cystoscopic biopsy was not advised as a compulsive process during cystoscopy, it was commonly utilized to develop treatment strategies in certain regions [10], particularly in some economically underdeveloped countries. Not only may a cystoscopic biopsy be used to determine the histologic grade preliminarily, but it can also be performed under local anaesthesia avoiding the unnecessary morbidity associated with general and spinal anaesthesia, reducing potential tumor dissemination and additional cost burden. Additionally, specifying the tumor grade aids in the treatment planning, as different tumor grades have varying risks of muscle invasion [11]. Therefore, accurate preoperative histologic grade determination is critical for therapeutic decision-making.

However, the histologic grade of current preoperative cystoscopic biopsy was frequently underestimated when compared to postoperative pathology [12], which may explain why it has not been widely used during cystoscopy. Besides, pathological grading classifications system has its own indeterminacy. Grade 2 is well recognized as a highly heterogeneous category in the WHO 1973 grading system, with reported proportion of bladder tumors classified as grade 2 ranging from 13 to 69% [13]. The use of WHO 2004 grading system is also recommended since it appears to be a more accurate predictor of tumor progression in clinical practice [14]. Nowadays severity of clinical symptoms [15], Vesical Imaging Reporting and Data System score (VI-RADS) [16] and endoscopic morphological features [17] could be helpful in determining tumor stage. Diagnostic ureteroscopic biopsy for upper urinary tract urothelial carcinoma could be helpful in prognostic analysis [18]. However, the prediction of pathological grade in cystoscopic biopsy may be related to certain factors that are still unknown.

In present study, we aimed to combine preoperative cystoscopic features and biopsy with clinical characteristics in order to accurately predict postoperative pathological grade, which could aid in early accurate diagnosis and treatment planning.

## Methods

### Ethics statement

The Ethics Committee of the First Affiliated Hospital of Nanjing Medical University (China) approved this study, and all participants provided informed written consent.

### Study population and general information

**Fig. 1:**
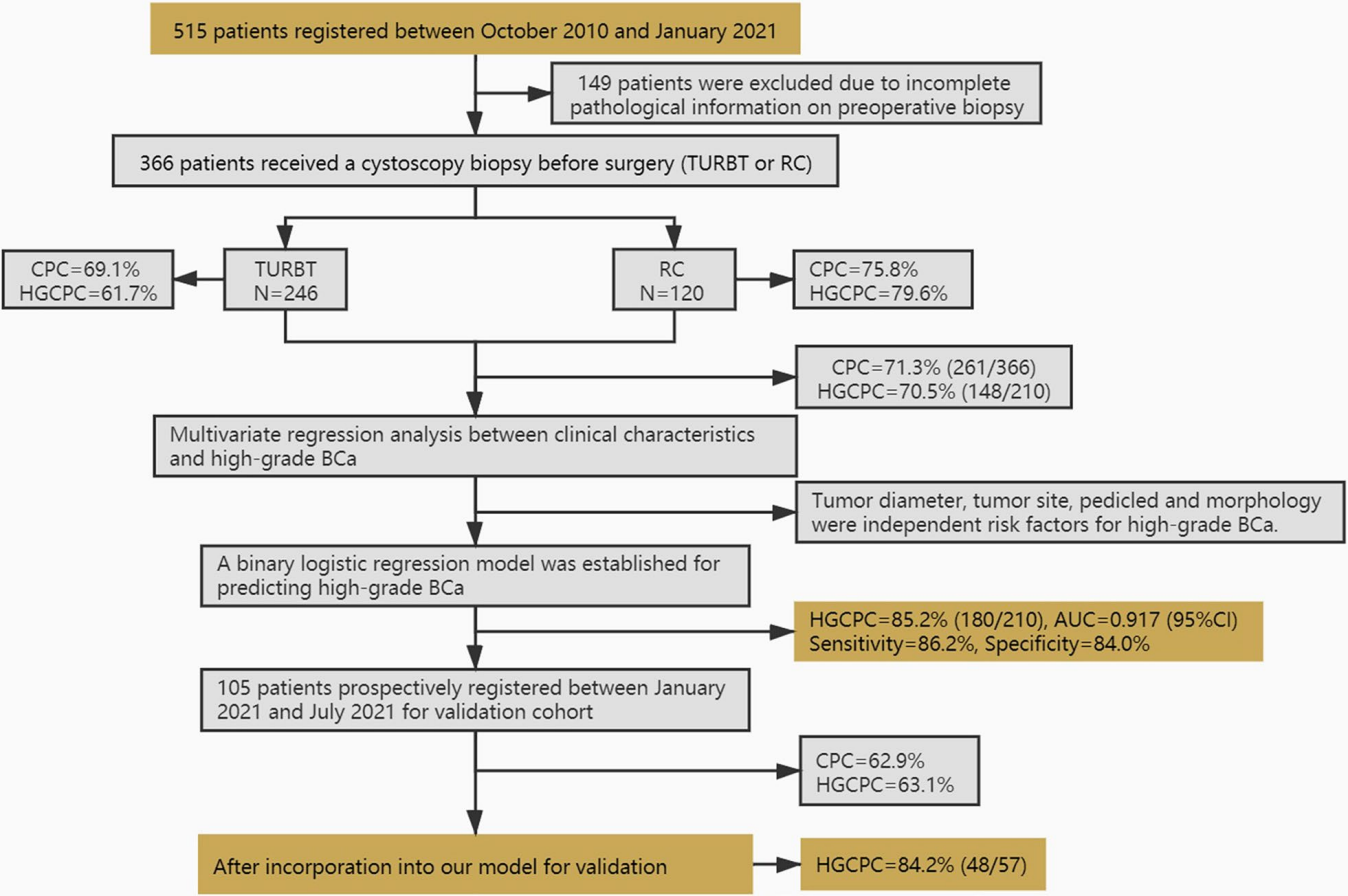
Flow chart of the study. BCa = bladder cancer; TURBT = transurethral resection of bladder tumor; RC = radical cystectomy; CPC = clinical pathology coincidence; HGCPC = high-grade clinical pathology coincidence

515 patients were included between October 2010 and January 2021, 149 patients were excluded due to incomplete pathological information on preoperative biopsy (ie, patients with unclear cystoscopic pictures; patients with no surgery within 1 month after biopsy; patients with chemotherapy or immunotherapy after biopsy). Then, the remaining 366 patients who meet the inclusion criteria were subsequently included in the study. All bladder biopsies were performed in outpatient setting. For training cohort, we retrospectively collected data on 366 patients who underwent TURBT or RC following cystoscopic biopsy for urothelial carcinoma at our centre between October 2010 and January 2021. Another 105 patients were included in the validation cohort between January 2021 and July 2021. All cases must be operated on immediately after the biopsy without any other treatment. If multiple biopsies were available for the same case, only the specimen closest to the operation is reviewed in this study. Clinical and pathological characteristics of training and validation cohort, including age, gender, smoking status, incidence frequency, tumor diameter, tumor number, tumor site and tumor morphology were respectively recorded and summarized in Table 1. We defined smokers as those who have smoked continuously or accumulatively for six months or more in their lifetime. Tumors were graded according to the WHO (2004) grading system [19], we used H as the high grade, L as the low grade, P as the low malignant potential, and G0 as the benign lesion including inflammation or atypical hyperplasia. Two urologic pathologists reviewed the tumor pathology and any discrepancies were resolved through consultation between the two pathologists. The study design is summarized in Fig. 1.

**Table 1:** Clinicopathological characteristics of bladder cancer patients in training and validation cohort

Variables	Number of patients (%)		*P* value
	Training cohort (n = 366)	Validation cohort (n = 105)	
Surgery option			**< 0.001**
TURBT	246 (67.2)	89 (84.8)	
RC	120 (32.8)	16 (15.2)	
Age			0.464
< 65	163 (44.5)	51 (48.6)	
≥ 65	203 (55.5)	54 (51.4)	
Gender			0.624
Female	85 (23.2)	22 (17.6)	
Male	281 (76.8)	83 (82.4)	
Incidence			0.281
Incipient	230 (62.8)	72 (68.6)	
Recurrent	136 (37.2)	33 (31.4)	
Smoking			0.196
No	274 (74.9)	85 (81)	
Yes	92 (25.1)	20 (19)	
Tumor diameter			0.338
< 3 cm	245 (66.9)	65 (61.9)	
≥ 3 cm	121 (33.1)	40 (38.1)	
Tumor number			0.689
Single	151 (39.3)	41 (39)	
Multiple	215 (60.7)	64 (61)	
Tumor pedicled			0.179
Yes	144 (38.9)	49 (46.7)	
No	222 (61.1)	56 (53.3)	
Tumor site			0.064
Single wall	215 (58.7)	51 (48.6)	
Multiple wall	151 (41.3)	54 (51.4)	
Tumor morphology			0.305
Cauliflower	193 (52.7)	49 (46.7)	
Seaweed	23 (6.3)	11 (10.5)	
Flat nipple	47 (12.8)	13 (12.4)	
Muscus	25 (6.8)	4 (3.8)	
Mixture	78 (21.3)	28 (26.7)	

The numbers marked in bold are statistically significant, *p* < 0.05

### Cystoscopic characteristics and histological grading criterions

All patients’ cystoscopic appearances were reviewed by two experienced urologists and any discrepancies were resolved through consultation between the two urologists. At least two tumor tissues were obtained during biopsy to avoid the difficulties with pathological classification due to small tissue sample size. Clinicopathological parameters were compared both between the cystoscopic biopsy grading and TURBT or RC pathological grading.

**Fig. 2:**
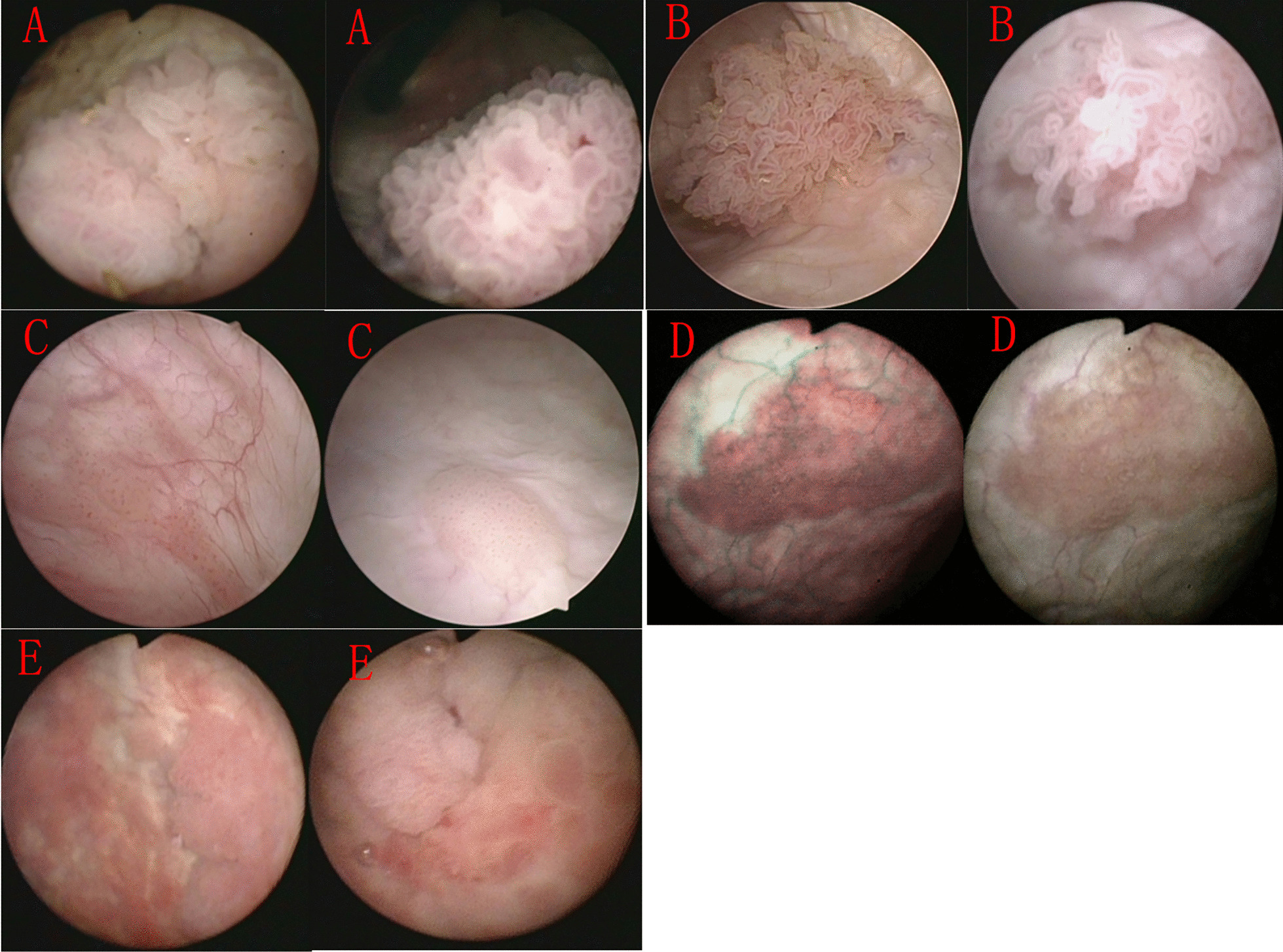
Typical morphology image of each category for bladder cancer. **A** Cauliflower morphology for bladder cancer. **B** Seaweed morphology for bladder cancer. **C** Flat nipple morphology for bladder cancer. **D** Muscus morphology for bladder cancer. **E** Mixture morphology for bladder cancer

We artificially classified cystoscopic morphology into the following five categories based on our experience: cauliflower pattern, seaweed pattern, flat nipple pattern, muscus pattern, and mixed pattern ((contained more than two forms)). Typical morphology image of each category was also provided (Fig. 2). Cauliflower morphology tumor is comparable to organic cauliflower consumption. Seaweed morphology tumor resembles coral seaweed in the ocean. A tumor with flat papillary morphology resembles the mushroom cap. Muscus morphology tumors like moss growing on a wall. The mixed sample contains more than two distinct types.

### Statistical analysis

The chi-square test was used to evaluate the association of patient information and cystoscopy characteristics with the final pathology grade. A multi-parameter diagnostic model for the postoperative pathological high-grade BCa was constructed by multivariable binary logistic regression analysis. All the above-mentioned statistical calculations were performed using SPSS 26.0 software (IBM Corp., Armonk, NY, USA). Receiver operating characteristic (ROC) curve, the areas under the ROC curve (AUC), 95% confidence interval (CI), sensitivity, specificity, and multi-parameter diagnostic model was produced using Graphpad Prism 8.3.0 software (GraphPad, San Diego, CA, USA). The forest plot of the risk factors in predicting pathological high-grade BCa was performed using R software (Version 4.0.3). All reported *P* values are 2-sided with statistical significance considered at ≤ 0.05.

## Results

### Clinical characteristics and BCa pathology

We included 366 patients who underwent TURBT or RC following cystoscopic biopsy for training cohort and another 105 patients for validation cohort (Table 1). Of the training cohort, 246 (67.2%) underwent TURBT following cystoscopic biopsy while 120 (32.8%) underwent RC following cystoscopic biopsy. When clinical pathology was considered, 261 (71.3%) had a biopsy pathology grade that was consistent with postoperative pathology grade. The histologic grade of 72 (19.7%) was upgraded, the histologic grade of 33 (9.0%) was downgraded. The association between coincidence rate and clinical characteristics was shown in Table 2. Age, incidence, tumor diameter, tumor number, tumor site and tumor morphology were found to be associated with pathological grading inconsistency. Subgroup analysis of TURBT and RC group’s grading conformance was shown in Additional file 1: Tables S1, S2.

**Table 2:** Association between clinicopathological characteristics and consistency in pathological grading pre- and postoperatively

Variables	Cases n (%)	Pathological grade consistency			*P* value
		Consistent	Downgrading	Upgrading	
All cases	366	261 (71.3)	33 (9.0)	72 (19.7)	
Age					**0.027**
< 65	163 (44.5)	119 (73.0)	20 (12.3)	24 (14.7)	
≥ 65	203 (55.5)	142 (70.0)	13 (6.4)	48 (23.6)	
Gender					0.337
Female	85 (23.2)	57 (67.1)	11 (12.9)	17 (20.0)	
Male	281 (76.8)	204 (72.6)	22 (7.8)	55 (19.6)	
Incidence					**0.001**
Incipient	230 (62.8)	174 (75.6)	11 (4.8)	45 (19.6)	
Recurrent	136 (37.2)	87 (64.0)	22 (16.2)	27 (19.8)	
Smoking					0.679
No	274 (74.9)	198 (72.3)	25 (9.1)	51 (18.6)	
Yes	92 (25.1)	72 (68.5)	8 (8.7)	23 (22.8)	
Tumor diameter					**0.009**
< 3 cm	245 (66.9)	169 (69.0)	30 (12.2)	46 (18.8)	
≥ 3 cm	121 (33.1)	92 (76.0)	3 (2.5)	26 (21.5)	
Tumor number					** < 0.001**
Single	151 (39.3)	105 (69.5)	24 (15.9)	22 (14.6)	
Multiple	215 (60.7)	156 (72.5)	9 (4.2)	50 (23.3)	
Tumor pedicled					0.169
Yes	144 (38.9)	98 (68.1)	18 (12.5)	28 (19.4)	
No	222 (61.1)	163 (73.4)	15 (6.8)	44 (19.8)	
Tumor site					**0.021**
Single wall	215 (58.7)	153 (71.2)	26 (12.1)	36 (16.7)	
Multiple wall	151 (41.3)	108 (71.5)	7 (4.6)	36 (23.8)	
Tumor morphology					**0.003**
Cauliflower	193 (52.7)	134 (69.4)	14 (7.3)	45 (23.3)	
Seaweed	23 (6.3)	17 (73.9)	5 (21.7)	1 (4.3)	
Flat nipple	47 (12.8)	31 (66.0)	7 (14.9)	9 (19.1)	
Muscus	25 (6.8)	19 (76.0)	5 (20.0)	1 (4.0)	
Mixture	78 (21.3)	60 (76.9)	2 (2.6)	16 (20.5)	

The numbers marked in bold are statistically significant, *p* < 0.05

### Association between clinical characteristics and postoperative high-grade BCa

Among the 366 patients in training cohort, 210 patients were confirmed as high-grade tumors postoperatively. The association between clinical characteristics and risk of developing high-grade tumor was shown in Table 3. We discovered that a larger tumor diameter (> 3 cm), multiple tumor numbers (≥ 2), older age (≥ 65 years-old), non-pedicled tumor, multiple tumor sites (≥ 2 sties) and tumor morphology were all associated with a high-grade tumor. The risk factors of developing high-grade tumor in TURBT and RC group were stratified analyzed in Additional file 1: Tables S3, S4.

**Table 3:** Correlations between the postoperative pathological grading and clinicopathological characteristics in BCa patients

Variables	Case n (%)	Postoperative Pathological Grade		*P* value
		Non-high grade	High grade	
All cases	366	156 (42.6)	210 (57.4)	
Age				**< 0.001**
< 65	163 (44.5)	91 (55.8)	72 (44.2)	
≥ 65	203 (55.5)	65 (32.0)	138 (68.0)	
Gender				0.232
Female	85 (23.2)	41 (48.2)	44 (51.8)	
Male	281 (76.8)	115 (40.9)	166 (59.1)	
Incidence				0.271
Incipient	230 (62.8)	93 (40.4)	137 (59.6)	
Recurrent	136 (37.2)	63 (46.3)	73 (53.7)	
Smoking				0.848
No	274 (74.9)	116 (42.3)	158 (57.7)	
Yes	92 (25.1)	40 (43.5)	52 (56.5)	
Tumor size				**< 0.001**
< 3 cm	245 (66.9)	126 (51.4)	119 (48.6)	
≥ 3 cm	121 (33.1)	30 (24.8)	91 (75.2)	
Tumor number				**< 0.001**
Single	151 (39.3)	88 (58.3)	63 (41.7)	
Multiple	215 (60.7)	68 (31.6)	147 (68.4)	
Tumor pedicled				**< 0.001**
Yes	144 (38.9)	96 (67.5)	48 (32.5)	
No	222 (61.1)	60 (27.0)	162 (73.0)	
Tumor site				**< 0.001**
Single wall	215 (58.7)	116 (54.0)	99 (46.0)	
Multiple wall	151 (41.3)	40 (26.5)	111 (73.5)	
Tumor morphology				**< 0.001**
Cauliflower	193 (52.7)	85 (44.0)	108 (56.0)	
Seaweed	23 (6.3)	22 (95.7)	1 (4.3)	
Flat nipple	47 (12.8)	23 (48.9)	24 (51.1)	
Muscus	25 (6.8)	16 (64.0)	9 (36.0)	
Mixture	78 (21.3)	10 (12.8)	68 (87.2)	

The numbers marked in bold are statistically significant, *p* < 0.05

### Univariate and multivariate regression analysis between clinical characteristics and high-grade BCa

Among the 210 patients with postoperative high-grade tumor in training cohort, only 70.5% (148/210) cases had the same pathological grade at biopsy. Univariate analysis revealed a correlation between postoperative high-grade BCa and age, tumor diameter, tumor number, tumor site, pedicled and morphology. On multivariate analysis, postoperative high-grade BCa was associated with larger tumor diameter (HR 3.056, *P* = 0.002), multiple tumor site (HR 2.927, *P* = 0.001), non-pedicled (HR 4.280, *P* < 0.001), seaweed morphology (HR 0.093, *P* = 0.029), muscus morphology (HR 0.113, *P* = 0.001), and biopsy-confirmed high-grade tumor (HR 36.745, *P* < 0.001) (Table 4).

**Table 4:** Univariate and multivariate binary logistic regression analysis of clinicopathological characteristics in predicting high-grade BCa

Variables	Univariate		Multivariate	
	HR (95% CI)	*P* value	HR (95% CI)	*P* value
Tumor diameter (≥ 3 cm vs. < 3 cm)	3.212 (1.982–5.205)	**< 0.001**	3.056 (1.529–6.110)	**0.002**
Tumor Site (Multiple walls vs. Single wall)	3.252 (2.073–5.100)	**< 0.001**	2.927 (1.518–5.642)	**0.001**
Tumor Pedicled (Non-pedicled vs. Pedicled)	5.400 (3.423–8.518)	**< 0.001**	4.280 (2.059–8.899)	**< 0.001**
Tumor morphology				
(Seaweed vs. Cauliflower)	0.036 (0.005–0.271)	**0.001**	0.093 (0.011–0.785)	**0.029**
(Flat nipple vs. Cauliflower)	0.821 (0.434–1.555)	0.546	0.514 (0.198–1.339)	0.173
(Muscus vs. Cauliflower)	0.443 (0.186–1.051)	0.065	0.113 (0.033–0.390)	**0.001**
(Mixture vs. Cauliflower)	5.352 (2.600–11.018)	** < 0.001**	2.089 (0.811–5.376)	0.127
Cystoscopic biopsy pathology				
(High-grade vs. G0)	33.188 (14.081–78.223)	** < 0.001**	36.745 (13.385–100.872)	**< 0.001**
(Low-grade vs. G0)	1.743 (0.809–3.753)	0.135	2.016 (0.804–5.056)	0.135
(Low malignant potential vs. G0)	1.281 (0.446–3.686)	0.272	2.089 (0.562–7.766)	0.272
Age (≥ 65 vs. < 65)	2.683 (1.750–4.113)	** < 0.001**	1.747 (0.931–3.280)	0.082
Tumor Number (Multiple vs. Single)	3.020 (1.959–4.655)	** < 0.001**	1.286 (0.568–2.914)	0.546
Gender (Male vs. Female)	1.345 (0.826–2.190)	0.233		
Incidence (Recurrent vs. Incipient)	0.787 (0.513–1.206)	0.271		
Smoking (Yes vs. No)	0.954 (0.592–1.538)	0.848		

The numbers marked in bold are statistically significant, *p* < 0.05

### Establishment of a multi-parameter diagnostic model for predicting postoperative high-grade BCa

**Fig. 3:**
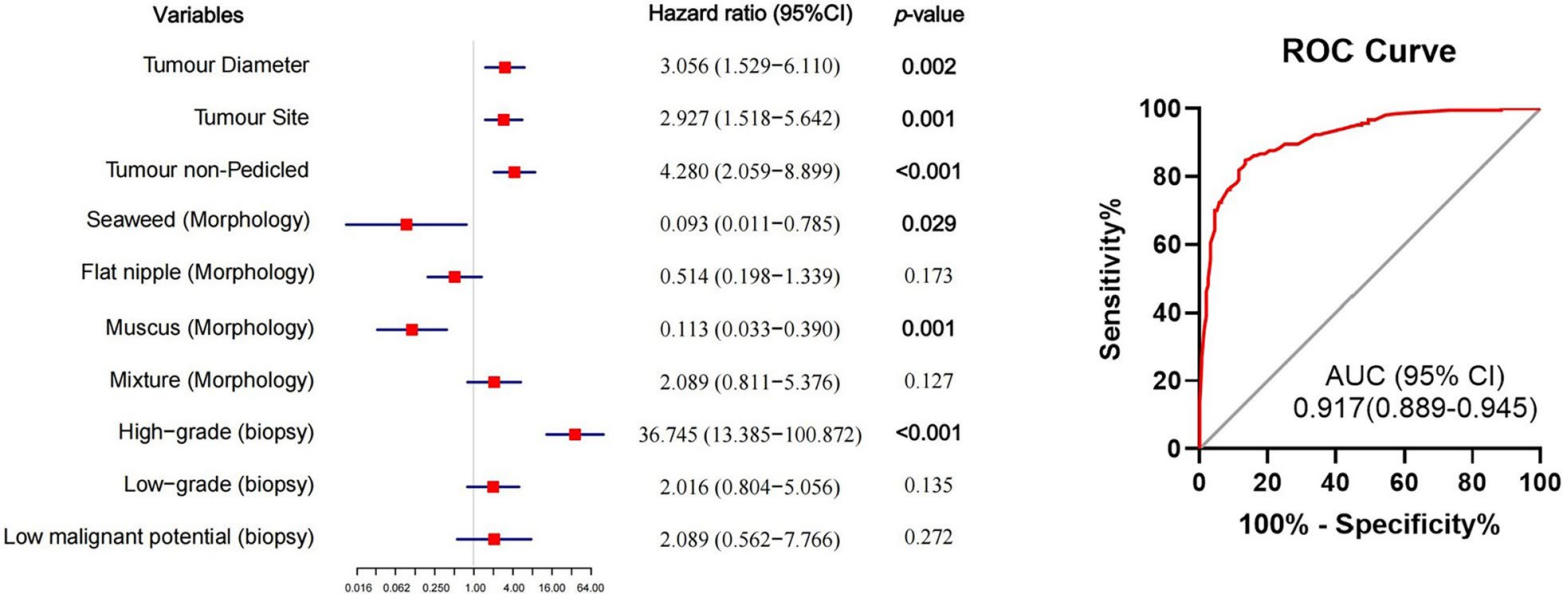
Forest plot of binary logistic regression analysis in BCa and ROC curve analysis for the “JSPH model” diagnostic performance

The multi-parameter diagnostic model of the five parameters for predicting postoperative high-grade BCa was constructed by multivariate regression analysis. We designated the predictive model as “JSPH model” and the equation of JSPH model was as follows: In [p/(1 − p)] = − 2.675 + 1.117 × tumor diameter + 1.074 × tumor site + 1.454 × non-pedicled − 2.371 × seaweed morphology − 2.082 × muscus morphology − 0.665 × flat nipple morphology + 0.736 × mixture morphology + 3.604 × high-grade biopsy pathology + 0.701 × low-grade biopsy pathology + 0.737 × low-malignant potential biopsy pathology (Fig. 3).

### Diagnostic performance of the JSPH model for high-grade BCa prediction

The diagnostic performance of the JSPH model for high-grade BCa conferred 86.2% sensitivity and 84.0% specificity. The AUC for the diagnostic performance of JSPH model was 0.917 (95% CI 0.889–0.945, *P* < 0.001), ROC curve analysis for the JSPH model diagnostic performance was summarized in Fig. 3. The JSPH model’s cut-off value for high-grade postoperative pathology was 0.5, which was defined by the binary logistic regression model. The JSPH model demonstrated high accuracy in detecting postoperative high-grade pathology in the training cohort, with a consistency rate of 85.2%.

### Prospective validation of JSPH model

After establishing the model, we prospectively collected 105 BCa cases for validation (Additional file 1: Table S5). In validation cohort, 89 (84.8%) patients underwent TURBT following cystoscopic biopsy and 16 (15.2%) patients underwent RC following cystoscopic biopsy. 62.9% (66/105) of these patients had biopsy pathology grade that were consistent with postoperative pathology grades (Additional file 1: Table S6). The association between clinical characteristics and risk of developing high-grade tumor in prospective cohort was shown in Additional file 1: Table S7.

Among the 105 patients, 57 had high-grade postoperative pathology. However, only 63.1% (36/57) cases exhibit consistency between biopsy pathology and postoperative pathology. After incorporation into our JSPH model for validation, the accuracy of predicting postoperative high-grade pathology was 84.2% (48/57).

## Discussion

We established a model which could enhance the diagnostic accuracy of high-grade tumor (85.2%) using cystoscopic biopsy prior to surgery. In a prospective validation group, the model also demonstrated a high level of accuracy of 84.2%. Tumor diameter, tumor site, non-pedicled, high-grade biopsy pathology, seaweed and muscus morphology were all independent risk factors for high-grade BCa.

There was a significant underestimation of pathology grade in preoperative cystoscopic biopsy. In our study, the pathological coincidence rate between preoperative cystoscopic biopsy and postoperative pathology was only 71.3% (261/366). Only 148 (70.5%) of the 210 postoperative high-grade cases were consistent with preoperative cystoscopic biopsy pathology. Possible explanations include the small size of biopsy tissues and the difficulty of biopsy due to the patient's low tolerance for cystoscopy. Besides, the biopsy tissue could not accurately reflect the entire tumor’s characteristics due to the tumor’s intrinsic heterogeneity. In order to overcome the cystoscopic defect in biopsy pathology, it had been reported that imaging methods such as CT (computed tomography) prediction, MRI (magnetic resonance imaging) and Micro Ultrasound were used to predict and improve the accuracy of grading or staging classification [20–22]. However, the prediction models built on imbalanced data are prone to overrepresent the majority type (high-grade) and underrepresent the minority type (low-grade), resulting in performing bias.

The morphology of bladder tumors varies significantly, and systematic definitions of the morphology have been lacking. Currently, it had been reported that the morphology of bladder tumors was associated with postoperative pathological stage [17]. In our study, we defined five distinct morphology subtypes and discovered that more than 70% of bladder tumors had cauliflower morphology. However, differentiation differences led to the development of various morphologies. Seaweed patterns were generally shallow, rarely infiltrated, and better differentiated than cauliflower patterns. Besides, seaweed and muscus morphologies had a higher rate of low-grade tumor while mixture morphology had a higher rate of high-grade tumor. However, mixture morphology was usually infiltrative, non-pedicle with a broad base and performed as high-grade tumor, which could reflect the tumor intrinsic heterogeneity. According to EORTC risk stratification, bladder tumors with a maximum diameter larger than 3 cm were deemed a risk factor for tumor progression [23], which could correspond to our “high-grade” tumors. Patients with pedicled bladder masses were considered to have a VI-RADS score ≤ 2 in clinical [24]. Del Giudice et al. [25] also reported that VI-RADS score ≤ 2 was deemed as a clinical predictor of NMIBC. Regarding pedicled tumors, they usually grew slowly and exhibited little aggressive behavior. High-grade bladder tumor tended to have more genetic alterations and a higher mutational load [26], which may predispose them to multiple and multisite tumor.

It was of great importance to confirm the pathological grade before operation. According to the criteria of tumor risk stratification in the EAU NMIBC guidelines [27], although some high-grade tumor could be intermediate risk based on additional risk factors, the majority of high-grade NMIBC are high risk tumors. Urine cytology (UC) has a high sensitivity for G3 and high-grade tumours (84%), however, the low sensitivity in G1/LG tumours (16%) has limited its clinical application [27]. Our model has demonstraed a high degree of specificity and sensitivity in distinguishing high grade BCa from low grade. In our prospective validation group, the pathological coincidence rate between preoperative cystoscopic biopsy and postoperative pathology was only 62.9% (66/105). Only 63.2% (36/57) postoperative high-grade cases were consistent with preoperative cystoscopic biopsy pathology. The pathological coincidence rate increased form 63.2% (36/57) to 84.2% (48/57), when our prediction model was used. Besides, the majority of MIBC cases could be classified as high-grade, because low-grade tumors and papillary urothelial neoplasms of low malignant potential were uneasy to infiltrate the basal membrane [11]. Therefore, more attention should be directed to the muscular layer examination and random biopsy during TURBT in patients with a high-grade pathology predicted by the model.

Although diagnostic TURBT was recommended prior to RC in accordance with European Association of Urology Guidelines [28], we might consider RC directly in patients with ≥ cT2 stage and predicted high grade pathology. The current multiparametric MRI (mp-MRI) based VI-RADS is capable of accurately predict tumor stage. However, there was still some misjudgment in assessing the muscular invasion of bladder tumor. It was reported that the cases scored as VI-RADS 4 had MIBC in the range of 89.7% to 100%, whereas case scored VI-RADS 3 had MIBC in the range of 45%–66.7% [16, 25, 29]. Therefore, it will be more beneficial to combine preoperative grade prediction and stage prediction when guiding the selection of treatment plan. For instance, patients with a VI-RADS score of 3 or 4 and a low-grade bladder cancer biopsy should exercise caution when selecting a treatment plan. If the predicted pathology remains low grade, we have a higher chance of preserving the bladder due to the low risk of invasion (Additional file 2: Fig. S1). If, on the other hand, the predicted pathology was high grade, we could avoid the potential influence of preoperative low-grade biopsy on treatment strategy selection, allowing us to consider RC directly (Additional file 3: Fig. S2).

Along with the VI-RADS score, the cystoscopic Likert score may also be useful in predicting tumor stage of bladder cancer, but its accuracy of was still not high enough. A total of 42 participants classified as Likert 3–5 underwent TURBT or cystectomy with pathological staging available, confirming MIBC in 13 [17]. Notably, when Likert ≥ 3 is present in combination with a confirmed high-grade tumor, it may indicate an increased risk of muscular infiltration. On the one hand, preoperative biopsy-induced underestimation could be avoided. On the other hand, cystoscope could be used exclusively to perform an accurate preoperative evaluation of tumor stage and grade, which aided in the formulation of a treatment plan. In addition, molecular markers have been explored in the diagnosis, prognosis prediction and recurrence monitoring of bladder cancer [30–32]. And the possibility for precise preoperative staging and grading of bladder cancer has been demotrated by cystoscopic Likert score and our cystoscopic predictive model. Thus, the implementing of cytology information with potential markers could benefit in future noninvasive precise diagnosis in bladder cancer.

Apart from the upgraded cases, we also focus on some degraded cases. In our training cohort, only 9.0% (33/336) of patients were pathological downgraded. Additionally, 72.7% (24/33) of them were downgraded following the operation due to obvious burns and deformations of pathological tissues during the operation, resulting in the absence of postoperative pathological conditions. Even if there is no postoperative pathology in these patients, the postoperative intravesical infusion plan must be based on the biopsy pathology, emphasizing the critical importance of pathological accuracy in biopsy.

Regarding limitations, this study is a single-center investigation that lacks external validation from other institutions. Besides, the tumor morphology description is a subjective judgment that may introduce bias. A larger prospective study is required to confirm our preliminary results.

## Conclusion

To our knowledge, we firstly present a model that could accurately predict postoperative pathological high-grade tumor prior to operations. We also use the validation cohort to verify the model’s accuracy. Our predictive model may be used to alternate TURBT to guide the surgical selection and potentially reduce anesthesia and operation risks, as well as the economic burden on patients, associated with diagnostic TURBT.

## Supplementary Information

**Additional file 1**. **Supplementary Table 1.** Association between clinicopathological characteristics and consistency in pathological grading pre- and postoperatively (TURBT group). **Supplementary Table 2.** Association between clinicopathological characteristics and consistency in pathological grading pre- and postoperatively (RC group). **Supplementary Table 3.** Correlations between the postoperative pathological grading of TURBT and clinicopathological features in BCa patients. **Supplementary Table 4.** Correlations between the postoperative pathological grading of RC and clinicopathological features in BCa patients. **Supplementary Table 5.** Clinicopathological characteristics of bladder cancer patients in validation cohort. **Supplementary Table 6.** Association between clinicopathological characteristics and consistency in pathological grading pre- and postoperatively (validation cohort). **Supplementary Table 7.** Correlations between the postoperative pathological grading and clinicopathological characteristics in BCa patients (validation cohort).

**Additional file 2**. **Fig. S1.** Cystoscopic and MRI scans of a patient with low-grade BCa on cystoscopic biopsy, low-grade BCa predicted by the JSPH model, and low-grade T1 BCa pathology on TURBT.

**Additional file 3**. **Fig. S2.** Cystoscopic and MRI scans of a patient with low-grade BCa on cystoscopic biopsy, high-grade BCa predicted by the JSPH model, and high-grade T2 BCa pathology on TURBT.

## Data Availability

All data generated or analysed during this study are included in this published article [and its supplementary information files].

## Abbreviations

BCa: Bladder cancer
TURBT: Transurethral resection of bladder tumor
RC: Radical cystectomy
NMIBC: Non-muscle-invasive bladder cancer
TMT: Trimodal therapy
WHO: World Health Organization
BCG: Bacillus Calmette-Guérin
AUC: Area under the curve
ROC: Receiver operating characteristics
CT: Computed tomography
MRI: Magnetic resonance imaging
VI-RADS: Vesical Imaging Reporting and Data System
EAU: European Association of Urology
CI: Confidence interval
