# Supplementary material for: The application value of multi-parameter cystoscope in improving the accuracy of preoperative bladder cancer grading
Source: BMC Urol. 2022 Jul 18;22:111. doi: 10.1186/s12894-022-01054-z (PMC9295426; doi:10.1186/s12894-022-01054-z)
Supplement: Supplementary file 1 — Additional file 1. Supplementary Table 1. Association between clinicopathological characteristics and consistency in pathological grading pre- and postoperatively (TURBT group). Supplementary Table 2. Association between clinicopathological characteristics and consistency in pathological grading pre- and postoperatively (RC group). Supplementary Table 3. Correlations between the postoperative pathological grading of TURBT and clinicopathological features in BCa patients. Supplementary Table 4. Correlations between the postoperative pathological grading of RC and clinicopathological features in BCa patients. Supplementary Table 5. Clinicopathological characteristics of bladder cancer patients in validation cohort. Supplementary Table 6. Association between clinicopathological characteristics and consistency in pathological grading pre- and postoperatively (validation cohort). Supplementary Table 7. Correlations between the postoperative pathological grading and clinicopathological characteristics in BCa patients (validation cohort). [file 12894_2022_1054_MOESM1_ESM.docx]

**Supplementary Table 1：Association between clinicopathological characteristics and consistency in pathological grading pre- and postoperatively (TURBT group).**

| **Variables** | **Cases**  **n（%）** | **Pathological Grade Consistency** | | | ***P* value** |
| --- | --- | --- | --- | --- | --- |
|  |  | [**Consistent**](#/javascript:;) | **Downgrading** | **Upgrading** |  |
| All cases | 246 | 170 (69.1) | 26 (10.6) | 50 (20.3) |  |
| **Age** |  |  |  |  | **0.021** |
| <65 | 119 (48.4) | 87 (73.1) | 16 (13.4) | 16 (13.4) |  |
| ≥65 | 127 (51.6) | 83 (65.4) | 10 (7.9) | 34 (26.8) |  |
| **Gender** |  |  |  |  | 0.304 |
| Female | 59 (24.0) | 36 (61.0) | 8 (13.6) | 15 (25.4) |  |
| Male | 187 (76.0) | 134 (71.7) | 18 (9.6) | 35 (18.7) |  |
| **Incidence** |  |  |  |  | **0.009** |
| Incipient | 152 (61.8) | 112 (73.7) | 9 (5.9) | 31 (20.4) |  |
| Recurrent | 94 (38.2) | 58 (61.7) | 17 (18.1) | 19 (20.2) |  |
| **Smoking** |  |  |  |  | 0.759 |
| No | 184 (74.8) | 129 (70.1) | 18 (9.8) | 37 (20.1) |  |
| Yes | 62 (25.2) | 41 (66.1) | 8 (12.9) | 13 (21.0) |  |
| **Tumor Diameter** |  |  |  |  | 0.055 |
| <3 cm | 179 (72.8) | 121 (67.6) | 24 (13.4) | 34 (19.0) |  |
| ≥3 cm | 67 (27.2) | 49 (73.1) | 2 (3.0) | 16 (23.9) |  |
| **Tumor Number** |  |  |  |  | **0.003** |
| Single | 114 (46.3) | 79 (69.3) | 19 (16.7) | 16 (14.0) |  |
| Multiple | 132 (53.7) | 91 (68.9) | 7 (5.3) | 34 (25.8) |  |
| **Tumor Pedicled** |  |  |  |  | 0.815 |
| Yes | 130 (52.8) | 90 (69.2) | 15 (11.5) | 25 (19.2) |  |
| No | 116 (47.2) | 80 (69.0) | 11 (9.5) | 25 (21.6) |  |
| **Tumor Site** |  |  |  |  | **0.044** |
| Single wall | 162 (65.9) | 112 (69.1) | 22 (13.6) | 28 (17.3) |  |
| Multiple wall | 84 (34.1) | 58 (69.0) | 4 (4.8) | 22 (26.2) |  |
| **Tumor Morphology** |  |  |  |  | **0.002** |
| Cauliflower | 138 (56.1) | 91 (65.9) | 12 (8.7) | 35(25.4) |  |
| Seaweed | 21 (8.5) | 16 (76.2) | 5 (23.8) | 0 (0.0) |  |
| Flat nipple | 28 (11.4) | 17 (60.7) | 6 (21.4) | 5 (17.9) |  |
| Muscus | 20 (8.1) | 16 (80.0) | 3 (15.0) | 1 (5.0) |  |
| Mixture | 39 (15.9) | 30 (76.9) | 0 (0.0) | 9 (23.1) |  |

**Supplementary Table 2：Association between clinicopathological characteristics and consistency in pathological grading pre- and postoperatively (RC group).**

| **Variables** | **Cases**  **n（%）** | **Pathological Grade Consistency** | | | ***P* value** |
| --- | --- | --- | --- | --- | --- |
|  |  | [**Consistent**](#/javascript:;) | **Downgrading** | **Upgrading** |  |
| All cases | 120 | 91 (75.8) | 7 (5.8) | 22 (18.3) |  |
| **Age** |  |  |  |  | 0.546 |
| <65 | 44 (36.7) | 32 (72.7) | 4 (9.1) | 8 (18.2) |  |
| ≥65 | 76 (63.3) | 59 (77.6) | 3 (3.9) | 14 (18.4) |  |
| **Gender** |  |  |  |  | 0.138 |
| Female | 26 (21.7) | 21 (80.8) | 3 (11.5) | 2 (7.7) |  |
| Male | 94 (78.3) | 70 (74.5) | 4 (4.3) | 20 (21.3) |  |
| **Incidence** |  |  |  |  | 0.129 |
| Incipient | 78 (65.0) | 62 (79.5) | 2 (2.6) | 14 (17.9) |  |
| Recurrent | 42 (35.0) | 29 (69.0) | 5 (11.9) | 8 (19.0) |  |
| **Smoking** |  |  |  |  | 0.144 |
| No | 90 (75.0) | 69 (76.7) | 7 (7.8) | 14 (15.6) |  |
| Yes | 30 (25.0) | 22 (73.3) | 0 (0.0) | 8 (26.7) |  |
| **Tumor Diameter** |  |  |  |  | 0.268 |
| <3 cm | 66 (55.0) | 48 (72.7) | 6 (9.1) | 12 (18.2) |  |
| ≥3 cm | 54 (45.0) | 43 (79.6) | 1 (1.9) | 10 (18.5) |  |
| **Tumor Number** |  |  |  |  | 0.071 |
| Single | 37 (30.8) | 26 (70.3) | 5 (13.5) | 6 (16.2) |  |
| Multiple | 83 (69.2) | 65 (78.3) | 2 (2.4) | 16 (19.3) |  |
| **Tumor Pedicled** |  |  |  |  | **0.035** |
| Yes | 14 (11.7) | 8 (57.1) | 3 (21.4) | 3 (21.4) |  |
| No | 106 (88.3) | 83 (78.3) | 4 (3.8) | 19 (17.9) |  |
| **Tumor Site** |  |  |  |  | 0.566 |
| Single wall | 53 (44.2) | 41 (77.4) | 4 (7.5) | 8 (15.1) |  |
| Multiple wall | 67 (55.8) | 50 (74.6) | 3 (4.5) | 14 (20.9) |  |
| **Tumor Morphology** |  |  |  |  | 0.270 |
| Cauliflower | 55 (45.8) | 43 (78.2) | 2 (3.6) | 10 (18.2) |  |
| Seaweed | 2 (1.7) | 1 (50.0) | 0 (0.0) | 1 (50.0) |  |
| Flat nipple | 19 (15.8) | 14 (73.7) | 1 (5.3) | 4 (21.1) |  |
| Muscus | 5 (4.2) | 3 (60.0) | 2 (40.0) | 0 (0.0) |  |
| Mixture | 39 (32.5) | 30 (76.9) | 2 (5.1) | 7 (17.9) |  |

**Supplementary Table 3**: **Correlations between the postoperative pathological grading of TURBT and clinicopathological features in BCa patients.**

| **Variables** | **Case**  **n (%)** | **Postoperative Pathological Grade** | | ***P* value** |
| --- | --- | --- | --- | --- |
|  |  | **Not-high grade** | **High grade** |  |
| All cases | 246 | 139 (56.5) | 107 (43.5) |  |
| **Age** |  |  |  | **<0.001** |
| <65 | 119 (48.4) | 83 (69.7) | 36 (30.3) |  |
| ≥65 | 127 (51.6) | 56 (44.1) | 71 (55.9) |  |
| **Gender** |  |  |  | 0.423 |
| Female | 59 (24.0) | 36 (61.0) | 23 (39.0) |  |
| Male | 187 (76.0) | 103 (55.1) | 84 (44.9) |  |
| **Incidence** |  |  |  | 0.618 |
| Incipient | 152 (61.8) | 84 (55.3) | 68 (44.7) |  |
| Recurrent | 94 (38.2) | 55 (58.5) | 39 (41.5) |  |
| **Smoking** |  |  |  | 0.992 |
| No | 184 (74.8) | 104 (56.5) | 80 (43.5) |  |
| Yes | 62 (25.2) | 35 (56.5) | 27 (43.5) |  |
| **Tumor Diameter** |  |  |  | **<0.001** |
| <3 cm | 179 (72.8) | 116 (64.8) | 63 (35.2) |  |
| ≥3 cm | 67 (27.2) | 23 (34.3) | 44 (65.7) |  |
| **Tumor Number** |  |  |  | **<0.001** |
| Single | 114 (46.3) | 80 (70.2) | 34 (29.8) |  |
| Multiple | 132 (53.7) | 59 (44.7) | 73 (55.3) |  |
| **Tumor Pedicled** |  |  |  | **<0.001** |
| Yes | 130 (52.8) | 88 (67.7) | 42 (32.3) |  |
| No | 116 (47.2) | 51 (44.0) | 65 (56.0) |  |
| **Tumor Site** |  |  |  | **<0.001** |
| Single wall | 162 (65.9) | 107 (66.0) | 55 (34.0) |  |
| Multiple wall | 84 (34.1) | 32 (38.1) | 52 (61.9) |  |
| **Tumor Morphology** |  |  |  | **<0.001** |
| Cauliflower | 138 (56.1) | 76 (55.1) | 62 (44.9) |  |
| Seaweed | 21 (8.5) | 21 (100.0) | 0 (0.0) |  |
| Flat nipple | 28 (11.4) | 21 (75.0) | 7 (25.0) |  |
| Muscus | 20 (8.1) | 14 (70.0) | 6 (30.0) |  |
| Mixture | 39 (15.9) | 7 (17.9) | 32 (82.1) |  |

**Supplementary Table 4**：**Correlations between the postoperative pathological grading of RC and clinicopathological features in BCa patients.**

| **Variables** | **Case**  **n (%)** | **Postoperative Pathological Grade** | | ***P* value** |
| --- | --- | --- | --- | --- |
|  |  | **Not-high grade** | **High grade** |  |
| All cases | 120 | 17 (14.2) | 103 (85.8) |  |
| **Age** |  |  |  | 0.337 |
| <65 | 44 (36.7) | 8 (18.2) | 36 (81.8) |  |
| ≥65 | 76 (63.3) | 9 (11.8) | 68 (88.2) |  |
| **Gender** |  |  |  | 0.403 |
| Female | 26 (21.7) | 5 (19.2) | 21 (80.8) |  |
| Male | 94 (78.3) | 12 (12.8) | 82 (87.8) |  |
| **Incidence** |  |  |  | 0.261 |
| Incipient | 78 (65.0) | 9 (11.5) | 69 (88.5) |  |
| Recurrent | 42 (35.0) | 8 (19.0) | 34 (81.0) |  |
| **Smoking** |  |  |  | 0.763 |
| No | 90 (75.0) | 12 (12.8) | 78 (86.7) |  |
| Yes | 30 (25.0) | 5 (16.7) | 25 (83.3) |  |
| **Tumor Diameter** |  |  |  | 0.732 |
| <3 cm | 66 (55.0) | 10 (15.2) | 56 (84.8) |  |
| ≥3 cm | 54 (45.0) | 7 (13.0) | 47 (87.0) |  |
| **Tumor Number** |  |  |  | 0.118 |
| Single | 37 (30.8) | 8 (21.6) | 29 (78.4) |  |
| Multiple | 83 (69.2) | 9 (10.8) | 74 (89.2) |  |
| **Tumor Pedicled** |  |  |  | **<0.001** |
| Yes | 14 (11.7) | 8 (57.1) | 6 (42.9) |  |
| No | 106 (88.3) | 9 (8.5) | 97 (91.5) |  |
| **Tumor Site** |  |  |  | 0.432 |
| Single wall | 53 (44.2) | 9 (17.0) | 44 (83.0) |  |
| Multiple wall | 67 (55.8) | 8 (11.9) | 69 (88.1) |  |
| **Tumor Morphology** |  |  |  | 0.116 |
| Cauliflower | 55 (45.8) | 9 (16.4) | 46 (83.6) |  |
| Seaweed | 2 (1.7) | 1 (50.0) | 1 (50.0) |  |
| Flat nipple | 19 (15.8) | 2 (10.5) | 17 (89.5) |  |
| Muscus | 5 (4.2) | 2 (40.0) | 3 (60.0) |  |
| Mixture | 39 (32.5) | 3 (7.7) | 36 (92.3) |  |

**Supplementary Table 5**: **Clinicopathological characteristics of bladder cancer patients in validation cohort.**

| **Variables** | **n (%)** |
| --- | --- |
| All cases | 105 |
| [**Surgery**](#/javascript:;) **Option** |  |
| TURBT | 89 (84.8) |
| RC | 16 (15.2) |
| **Age** |  |
| <65 | 51 (48.6) |
| ≥65 | 54 (51.4) |
| **Gender** |  |
| Female | 22 (17.6) |
| Male | 83 (82.4) |
| **Tumor Diameter** |  |
| <3 cm | 65 (61.9) |
| ≥3 cm | 40 (38.1) |
| **Tumor Pedicled** |  |
| Yes | 49 (46.7) |
| No | 56 (53.3) |
| **Tumor Site** |  |
| Single wall | 51 (48.6) |
| Multiple wall | 54 (51.4) |
| **Tumor Morphology** |  |
| Cauliflower | 49 (46.7) |
| Seaweed | 11 (10.5) |
| Flat nipple | 13 (12.4) |
| Muscus | 4 (3.8) |
| Mixture | 28 (26.7) |

**Supplementary Table 6**: **Association between clinicopathological characteristics and consistency in pathological grading pre- and postoperatively (validation cohort).**

| **Variables** | **Cases**  **n (%)** | **Pathological Grade Consistency** | | | ***P* value** |
| --- | --- | --- | --- | --- | --- |
|  |  | [**Consistent**](#/javascript:;) | **Downgrading** | **Upgrading** |  |
| All cases | 105 | 66 (62.9) | 4 (3.8) | 35 (33.3) |  |
| **Age** |  |  |  |  | 0.469 |
| <65 | 51 (48.6) | 33 (64.7) | 3 (5.9) | 15 (29.4) |  |
| ≥65 | 54 (51.4) | 33 (61.1) | 1 (1.9) | 20 (37.0) |  |
| **Gender** |  |  |  |  | 0.081 |
| Female | 22 (17.6) | 10 (45.5) | 2 (9.1) | 10 (45.5) |  |
| Male | 83 (82.4) | 56 (67.5) | 2 (2.4) | 25 (30.1) |  |
| **Tumor Diameter** |  |  |  |  | 0.433 |
| <3 cm | 65 (61.9) | 40 (61.5) | 4 (6.2) | 21 (32.3) |  |
| ≥3 cm | 40 (38.1) | 26 (65.0) | 0 (0.0) | 14 (35.0) |  |
| **Tumor Pedicled** |  |  |  |  | 0.096 |
| Yes | 49 (46.7) | 30 (61.2) | 4 (8.2) | 15 (30.6) |  |
| No | 56 (53.3) | 36 (64.3) | 0 (6.8) | 20 (35.7) |  |
| **Tumor Site** |  |  |  |  | 0.120 |
| Single wall | 51 (48.6) | 32 (62.7) | 4 (7.8) | 15 (29.4) |  |
| Multiple wall | 54 (51.4) | 34 (63.0) | 0 (0.0) | 20 (37.0) |  |
| **Tumor Morphology** |  |  |  |  | 0.163 |
| Cauliflower | 49 (46.7) | 35 (71.4) | 2 (4.1) | 12 (24.5) |  |
| Seaweed | 11 (10.5) | 5 (45.5) | 2 (18.2) | 4 (36.4) |  |
| Flat nipple | 13 (12.4) | 6 (46.2) | 0 (0.0) | 7 (53.8) |  |
| Muscus | 4 (3.8) | 2 (50.0) | 0 (0.0) | 2 (50.0) |  |
| Mixture | 28 (26.7) | 18 (64.3) | 0 (0.0) | 10 (35.7) |  |

**Supplementary Table 7**：**Correlations between the postoperative pathological grading and clinicopathological characteristics in BCa patients (validation cohort).**

| **Variables** | **Case**  **n (%)** | **Postoperative Pathological Grade** | | ***P* value** |
| --- | --- | --- | --- | --- |
|  |  | **Not-high grade** | **High grade** |  |
| All cases | 105 | 48 (45.7) | 57 (54.3) |  |
| **Age** |  |  |  | **0.003** |
| <65 | 51 (48.6) | 31 (60.8) | 20 (39.2) |  |
| ≥65 | 54 (51.4) | 17 (31.5) | 37 (68.5) |  |
| **Gender** |  |  |  | 0.611 |
| Female | 22 (17.6) | 9 (40.9) | 13 (59.1) |  |
| Male | 83 (82.4) | 39 (47.0) | 44 (53.0) |  |
| **Tumor Diameter** |  |  |  | **＜****0.001** |
| <3 cm | 65 (61.9) | 40 (61.5) | 25 (38.5) |  |
| ≥3 cm | 40 (38.1) | 30 (24.8) | 91 (75.2) |  |
| **Tumor Pedicled** |  |  |  | **＜0.001** |
| Yes | 49 (46.7) | 37 (75.5) | 12 (24.5) |  |
| No | 56 (53.3) | 11 (19.6) | 45 (80.4) |  |
| **Tumor Site** |  |  |  | **0.026** |
| Single wall | 51 (48.6) | 29 (56.9) | 22 (43.1) |  |
| Multiple wall | 54 (51.4) | 19 (35.2) | 35 (64.8) |  |
| **Tumor Morphology** |  |  |  | **＜0.001** |
| Cauliflower | 49 (46.7) | 26 (53.1) | 23 (46.9) |  |
| Seaweed | 11 (10.5) | 11 (100.0) | 0 (0.0) |  |
| Flat nipple | 13 (12.4) | 6 (46.2) | 7 (53.8) |  |
| Muscus | 4 (3.8) | 2 (50.0) | 2 (50.0) |  |
| Mixture | 28 (26.7) | 3 (10.7) | 25 (89.3) |  |
