# Supplementary figures and images for: The application value of multi-parameter cystoscope in improving the accuracy of preoperative bladder cancer grading
Source: BMC Urol. 2022 Jul 18;22:111. doi: 10.1186/s12894-022-01054-z (PMC9295426; doi:10.1186/s12894-022-01054-z)

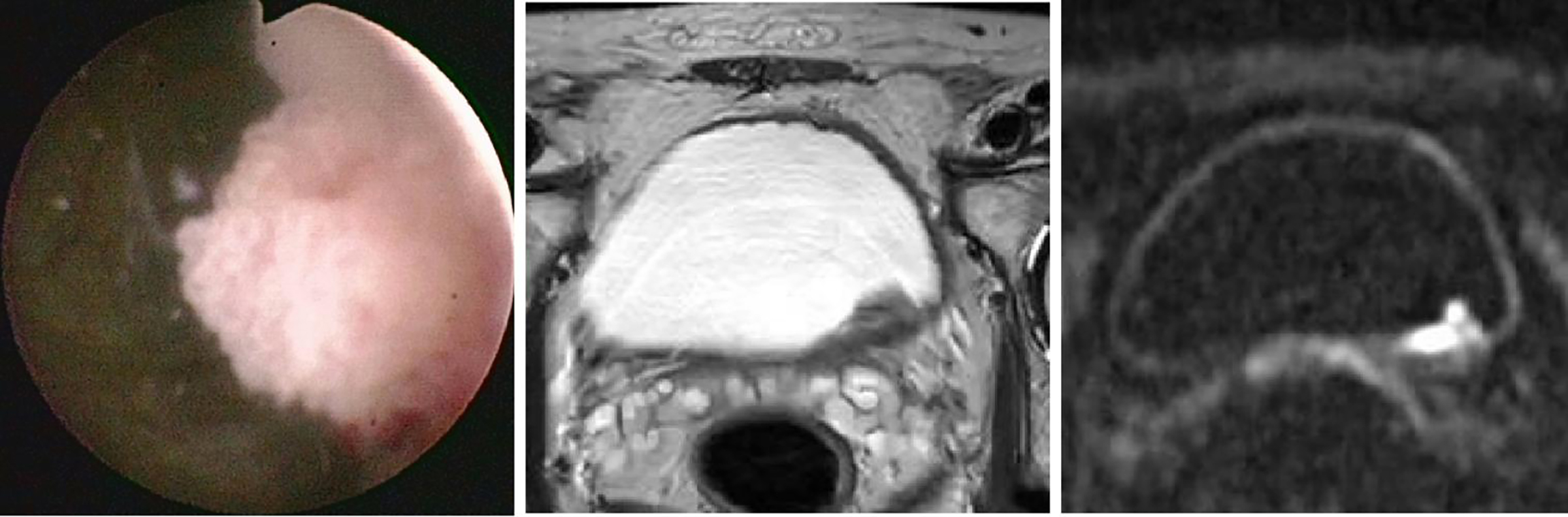

Supplement: Supplementary file 2 — Additional file 2. Fig. S1. Cystoscopic and MRI scans of a patient with low-grade BCa on cystoscopic biopsy, low-grade BCa predicted by the JSPH model, and low-grade T1 BCa pathology on TURBT. [file 12894_2022_1054_MOESM2_ESM.jpg]

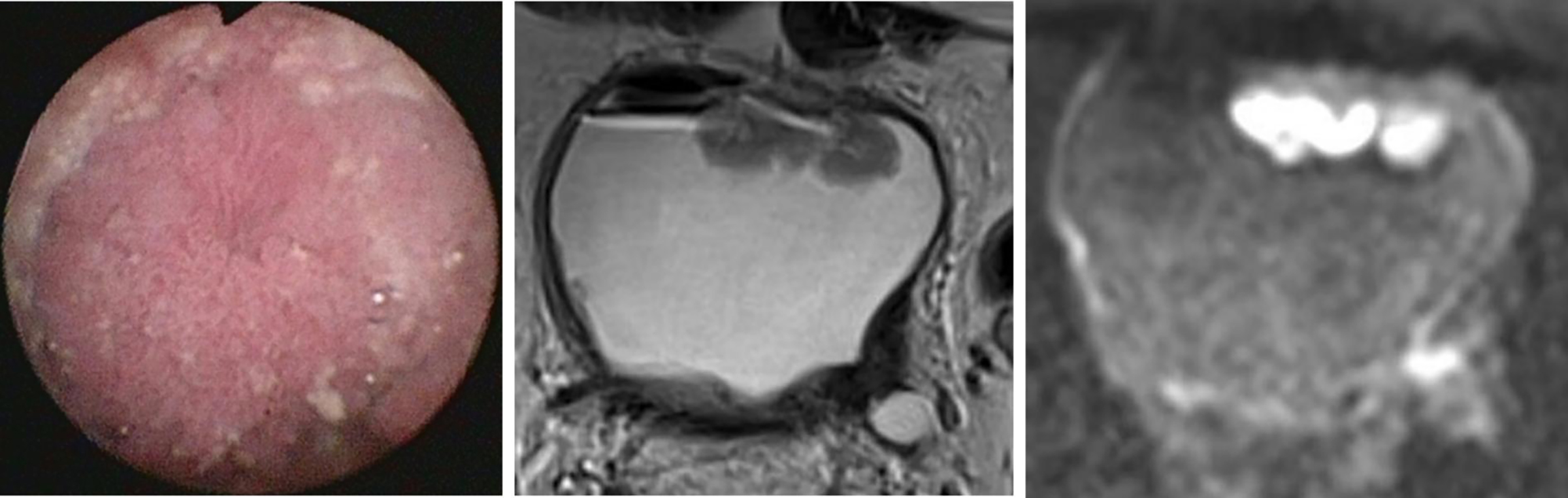

Supplement: Supplementary file 3 — Additional file 3. Fig. S2. Cystoscopic and MRI scans of a patient with low-grade BCa on cystoscopic biopsy, high-grade BCa predicted by the JSPH model, and high-grade T2 BCa pathology on TURBT. [file 12894_2022_1054_MOESM3_ESM.jpg]
